# Platelet Membrane: An Outstanding Factor in Cancer Metastasis

**DOI:** 10.3390/membranes12020182

**Published:** 2022-02-03

**Authors:** Nazly Z. Durán-Saenz, Alejandra Serrano-Puente, Perla I. Gallegos-Flores, Brenda D. Mendoza-Almanza, Edgar L. Esparza-Ibarra, Susana Godina-González, Irma E. González-Curiel, Jorge L. Ayala-Luján, Marisa Hernández-Barrales, Cecilia F. Cueto-Villalobos, Sharahy Y. Frausto-Fierros, Luis A. Burciaga-Hernandez, Gretel Mendoza-Almanza

**Affiliations:** 1 Biomedical Sciences, Autonomous University of Zacatecas, Zacatecas 98160, Mexico; 29101139@uaz.edu.mx (N.Z.D.-S.); 29106068@uaz.edu.mx (A.S.-P.); irmacuriel@uaz.edu.mx (I.E.G.-C.); jayala69@uaz.edu.mx (J.L.A.-L.); luis.burciaga@uaz.edu.mx (L.A.B.-H.); 2 Academic Unit of Biological Sciences, Autonomous University of Zacatecas, Zacatecas 98068, Mexico; ivonne_gf@uaz.edu.mx (P.I.G.-F.); brenda.mendoza@uaz.edu.mx (B.D.M.-A.); lesparza@uaz.edu.mx (E.L.E.-I.); 3 Academic Unit of Chemical Sciences, Autonomous University of Zacatecas, Zacatecas 98160, Mexico; sgodina@uaz.edu.mx (S.G.-G.); marisahb@uaz.edu.mx (M.H.-B.); 36170554@uaz.edu.mx (C.F.C.-V.); 36175355@uaz.edu.mx (S.Y.F.-F.); 4 National Council of Science and Technology, Autonomous University of Zacatecas, Zacatecas 98000, Mexico

**Keywords:** platelet membrane, cancer cell membrane, microenvironment, receptors

## Abstract

In addition to being biological barriers where the internalization or release of biomolecules is decided, cell membranes are contact structures between the interior and exterior of the cell. Here, the processes of cell signaling mediated by receptors, ions, hormones, cytokines, enzymes, growth factors, extracellular matrix (ECM), and vesicles begin. They triggering several responses from the cell membrane that include rearranging its components according to the immediate needs of the cell, for example, in the membrane of platelets, the formation of filopodia and lamellipodia as a tissue repair response. In cancer, the cancer cells must adapt to the new tumor microenvironment (TME) and acquire capacities in the cell membrane to transform their shape, such as in the case of epithelial−mesenchymal transition (EMT) in the metastatic process. The cancer cells must also attract allies in this challenging process, such as platelets, fibroblasts associated with cancer (CAF), stromal cells, adipocytes, and the extracellular matrix itself, which limits tumor growth. The platelets are enucleated cells with fairly interesting growth factors, proangiogenic factors, cytokines, mRNA, and proteins, which support the development of a tumor microenvironment and support the metastatic process. This review will discuss the different actions that platelet membranes and cancer cell membranes carry out during their relationship in the tumor microenvironment and metastasis.

## 1. Overview

The molecular organization of cell membranes has been characterized thanks to studies that have assessed their mobility. This characterization has provided evidence of functions performed by cell membranes depending on the distribution and interaction of lipids and the clustering of signaling proteins on their surface [1]. The dynamic exchange of biomolecules inside and outside the cell is explained as the result of the organization of proteins in the membrane, and their flux is related to intrinsic factors of the lipid bilayer [2], such as the presence of lipid rafts [3] and the tetraspanin network [4], as well as to extrinsic factors, such as cortical actin [5] and galectins [6], among others.

Cell membranes are affected by different physical or chemical disturbances that force them to change their shape, without affecting the cell integrity. The most critical component of the plasma membrane that supports these changes is the cortical filamentous actin cytoskeleton, which is located immediately below the plasma membrane. An essential role in this process is also played by the compartments formed by fence and picket proteins, which restrict the diffusion of membrane proteins and phospholipids [7,8]. The interaction between actin and membrane proteins plays an essential role in determining the general mechanical stiffness of the membrane and its organization [9].

During the metastatic process, significant changes occur in the membrane of cancer cells that give them the ability to migrate to sites distant from the primary tumor. These changes include the following: (1) Eliminating cell−cell junctions and the ECM by metastatic clones. The loss of these junctions triggers a process called anoikis, a type of cell death due to apoptosis that occurs as a result of the loss of cell−cell junctions [10,11]. (2) Metastatic cells acquire the ability to avoid anoikis by suppressing its pathways through the involvement of platelets [12]. (3) The expression of proteins related to the mesenchymal phenotype is activated, triggering the EMT [13]. (4) Metastatic cells become ready for intravasation into the microvessels generated by angiogenesis in the TME [14], developing the ability to attract and conglomerate platelets around them. Platelets provide metastatic cells with the protection they need to travel without being destroyed by the shear force generated by circulating blood or natural killer (NK) cells [15]. These so-called circulating tumor cells (CTC) can extravasate at a site distant from their point of origin, forming a new metastatic focus with the help of platelets [16].

Platelets are enucleated cells whose biogenesis begins with the fragmentation of megakaryocytes [17]. Now they are in the focus of several research groups worldwide due to their enormous potential to change our paradigmatic understanding of the development of several diseases. Platelets are currently an important focus of research due to their role in developing cancer, angiogenesis, and metastasis [15]. They have various membrane-bound organelles, including mitochondria, alpha granules, dense granules, and lysosomes, in addition to a complex membranous system, known as the open canalicular system (OCS), which allows for the connection of cytosol (as an external medium) and the dense tubular system (DTS), which stores essential metabolic enzymes [18].

Platelet mitochondria and DTS are responsible for generating the energy necessary for platelet activation and the release of granule content [19]. However, calcium is a necessary element to (1) trigger platelet activation [20]; (2) reorganize the platelet cytoskeleton during the shape changes in the activation [21,22], and (3) rearrange platelet surface receptors when adhering to other platelets or tumor cells, as well as to the subendothelial matrix [21,22]. The DTS and the OCS are in charge of providing the necessary calcium to the platelets [23]. These organelles store calcium and can rapidly discharge it into the platelet cytosol. The calcium release from these organelles leads to a profound ultrastructural change of the membrane, from an elongated and thin shape to a round vesicular shape [24].

## 2. Platelets

Platelets perform diverse functions in the body. They play a critical role in hemostasis, coagulation, thrombosis, interaction, and elimination of pathogens such as bacteria and viruses from the bloodstream [25]. In recent decades, surprising discoveries have been made regarding the ability of platelets to intervene in pathological processes such as autoimmune diseases [26], diabetes [27], hypertension [28], cancer [29,30], and COVID-19 [25,31,32].

**Figure 1:**
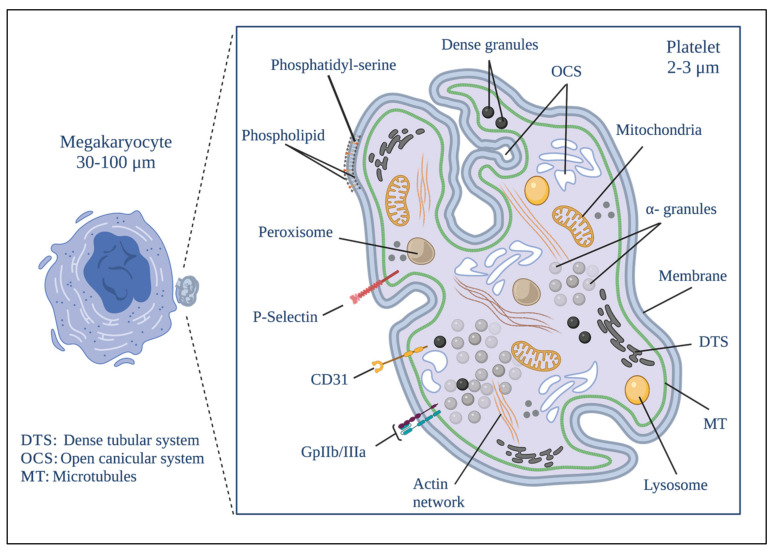
Platelet morphology. The biogenesis of platelets begins in the formation of protrusions in the megakaryocyte known as proplatelets. The biological material that will give rise to the platelet is deposited simultaneously to their formation after the fragmentation of the megakaryocyte proplatelet. Platelets develop a series of distinguishable structural elements that include a delimited plasma membrane; invaginations of the superficial membrane that form the open canicular system (OCS); a network of closed channels of the residual endoplasmic reticulum that forms the dense tubular system (DTS); a spectrin-based membrane backbone; an actin-based cytoskeletal network; a peripheral band of microtubules; and numerous organelles including alpha granules; dense granules; peroxisomes; lysosomes; mitochondria and numerous receptors on the different membranes that exist on the platelet. Created by BioRender.

Platelet biogenesis begins during the maturational stage of megakaryocytes [17], within a cytoplasmic cavity with pseudopod-like structures called proplatelets where proteins and organelles will later become part of the platelets that are deposited [33,34]. The rearrangement of platelet cytoplasmic structures also begins in this cavity. The development of the demarcation membrane system (DMS) occurs here. It is formed by an extensive network of membranous channels formed by flattened tubules and cisternae derived from tubular invaginations of the plasma membrane. This system is called the invaginated membrane system and is the origin of the proplatelet membrane [33,34,35,36]. The DMS allows both megakaryocytes and platelets to have more significant contact with the outside, and constitutes a membrane reservoir for forming and spreading proplatelets and platelets [35,36]. Another cytoplasmic structure that begins to form in the cytoplasmic cavity mentioned above is DTS, which constitutes the main reservoir of intracellular Ca^2+^ and the place where prostaglandins are synthesized. This system is not in contact with the outside and is derived from the endoplasmic reticulum [37,38]. Figure 1 shows the platelet structure.

Tubulin and actin are the main components of the cytoskeletal network of proplatelets [39]. Thanks mainly to the continuous polymerization of the tubulin bundles at their free ends and to the sliding of superimposed microtubules driven by dynein, proplatelets can change their shape by extending or contracting [40]. When proplatelets acquire the components of functional platelets, they are excised from the megakaryocyte and released into the bloodstream as platelets with a size ranging from 2 to 3 µm [17,33].

In platelets, the submembrane area, located immediately below the lipid bilayer, contains the contractile system of the platelet membrane (which is made up of actin filaments) that allows platelets to change shape and their receptors to translocate [41]. Platelets modulate the shape and size of their membrane through processes such as pseudopod formation, adhesion, and platelet aggregation [42]. Various cellular structures, biomolecules, and mechanisms participate in these processes, including (1) the membrane reservoir in the DMS that is formed from the biogenesis of platelets and that is used in the extension and degranulation activities associated with platelet activation [33,36]; (2) the actin cytoskeleton of platelets [22]; (3) the activation of Rac1 and the redistribution and activation of the integrin αIIbβ3, which allow cells to change shape, mediated by the membrane and giving the platelets greater flexibility when spreading to small sites of the exposed subendothelium. Rac1 is an essential factor in the release of fibronectin and fibrinogen from the granules when α is activated by integrins [43].

The circulating platelet population is heterogeneous in size, age, content, and responsiveness [44]. The latter characteristic has been studied in works focused on assessing the functional and structural differences between subpopulations of platelets concerning the variability of their response according to the different coding and non-coding RNAs within platelets. Platelet responsiveness is also affected by the different processing pathways of RNA transcripts, protein translation, and synthesis mechanisms, as well as other platelet biomolecules that regulate cellular activity such as coagulation factors, growth factors, chemokines, cytosines, microbicidal proteins, and prostaglandins, stored in the alpha, dense, and lysosome granules.

### 2.1. Platelet Granules

Alpha granules are the most abundant in platelets. A single platelet can contain between 50 and 80 [45]. Like all platelet granules, alpha granules are surrounded by a membrane. They have two main compartments, the nucleoid compartment, which contains a large number of proteoglycans, and the matrix compartment, which contains the plasma proteins and the proteins necessary for platelet activity [45,46,47], such as the von Willebrand factor (vWF), multimerin-1, and factor V [48]. Alpha granules are formed at the same time as the megakaryocyte reaches maturity. Some of the proteins of the alpha granules are synthesized in the megakaryocyte and rough endoplasmic reticulum, and are then packed into storage vesicles in the Golgi apparatus [49]. Other proteins come from liquid phase endocytosis events [50]. Thus, these granules store both membrane and soluble proteins [51]. During platelet activation, alpha granules secrete proteins involved in critical cellular functions. Most of them are related to cancer at the different stages of tumorigenesis, angiogenesis, or metastasis (we indicate with the superscript C, the platelet factors involved in cancer). (1) cell adhesion: vWF^C^, fibrinogen^C^, fibronectin^C^, vitronectin, TSP-1^C^, TSP-2, and laminin^C^ 8. (2) Growth and mitogenic factors: PDGF^C^, EGF-2^C^, HGF^C^, SCUBE1^C^, IGF-1^C^, IGFBP-3, VEGF^C^, FGF-2^C^, BMP-2^C^, BMP-4^C^, BMP-6^C^, BMP-11, GDF-15^C^, gremlin-1^C^, CTGF^C^. (3) Cytokines and chemokines: TGF-β1^C^, -β2, -β4, IL-1α^C^, IL-1β, TNF-α^C^, TNF-β^C^, IFNγ^C^, CCL2^C^, CCL3, CCL4^C^, CCL5 (RANTES)^C^, CCL7^C^, CCL14, CCL15^C^, CCL17^C^, CCL19^C^, CCL20^C^, CCL21^C^, CCL22^C^, CXCL1, CXCL2, CXCL3^C^, CXCL4 (PF4)^C^, CXCL4L1^C^, CXCL5^C^, CXCL6^C^, NAP-2, CXCL7^C^, CXCL8 (IL-8)^C^, CXCL10^C^, CXCL11^C^, CXCL12 (SDF-1α)^C^, GM-CSF, CSF-1^C^, CXCL16^C^, TNFSF14, TPO, ANG-1^C^, ANG-2^C^, ANGLP 2, HMGB1^C^, IL-6sR, osteonectin^C^, BSP^C^, Dkk1^C^, Wnt3a^C^, osteoprotegerin^C^, BDNF^C^, and γ-interferon protein-10 [45,46,47,48,49,50,51,52]. Each granule contains proteins with opposite activities; for example, they contain (1) pro- and anti-coagulants: Clotting factors and inhibitors: Factor (F) V/Va^C^, FVI, FVII^C^, FVIII^C^, FX, FXI, FXIIIa, prothrombin^C^, multimerin 1, protease nexin-1, nexin-2, TFPI^C^, PCI^C^. (2) Proteases and protease inhibitors: metalloproteinases (MMP) MMP-1^C^, MMP-2^C^, MMP-3, MMP-4, MMP-9^C^, MMP-14^C^, ADAMTS-13^C^, ADAM-10^C^, ADAM-17, TIMP1^C^, TIMP2, TIMP3^C^, C1 inhibitor, α1-antitrypsin, α2-antitrypsin, α2-macroglobulin, granzyme B, and bradykinin [45,46,47,48,49,50,51,52].

The membrane of the alpha granules contains unique molecular receptors whose ligand recognition site is oriented towards their inner side, and will only be exposed when the platelet has been activated. Several of the platelet proteins and receptors participate in some fundamental stages of cancer development and spread (Table 1). Some of these receptors, such as P-selectin^C^, osteonectin^C^, and GMP-33, are specific receptors that are absent from the plasma membrane of resting platelets [52,53,54,55,56,57,58,59,60,61,62,63]. They also contain several dozen angiogenesis regulatory proteins that function as mobile growth regulators of new blood vessels [60,61].

Dense granules are the most minuscule granules in platelets. GTP (guanosine-5′-triphosphate), Ral (is a GTPase member of the Ras family, which have several effector molecules and are rapidly activated by an increase of intracellular Ca^2+^ levels), and Rab27^C^ (regulates dense granule secretion in platelets) binding proteins are found in the membrane of dense granules [64], Table 1. They contain simpler and smaller molecules than alpha granules, such as catecholamines, ADP, ATP, polyphosphate, and Ca^2+^ [63]. They are also rich in lysolecithin and GM3 ganglioside. They are delimited by a single membrane and have a dense nucleus.

Lysosomes come from megakaryocytes and are recognizable from the start of megakaryocyte biogenesis [65]. The lysosomal integral membrane protein LIMP (CD63)^C^; two distinct lysosome-associated membrane proteins, LAMP-1^C^ and LAMP-2^C^, which have a single transmembrane domain [66]; and a tetraspanin are found in the membrane of lysosomes [67]. These proteins are all are strongly glycosylated on the luminal side of the lysosome, forming a protective layer against the hydrolytic enzymes stored in the granule. Lysosomes contain several glycosidases, proteases, and cationic proteins with a bactericidal activity, such as βN-acetylglucosaminidase and βN-acetylgalactosaminidase β-glucuronidase, β-galactosidase, α-mannosidase, β-glycerophosphatase, arylsulfatase, α-arabinosidase, heparinase, endoglucosidase, cathepsin, collagenase, and elastase [68,69].

### 2.2. Mitochondria

Platelet mitochondria provide the energy necessary for platelets to perform their vital functions [70]. They have the same shape as any other mitochondrial membrane in any other cell in the organism. They are delimited by two concentric membranes, an outer and an inner membrane, with invaginations of the inner mitochondrial membrane into the interior of the mitochondrial matrix [70,71]. When the platelet is activated, mitochondria are released freely through a mechanism similar to the release of exosomes. They can also be released into vesicles [72,73]. Another pathway for the release of mitochondria by platelets is autophagy, a process that has been shown to occur in platelets, induced by platelet activation [74]. In type 2 diabetes, for example, platelet mitophagy, which is selective autophagy of damaged mitochondria, serves as a protective mechanism for platelets against oxidative stress, preventing apoptosis and preserving platelet functions [75,76].

### 2.3. Filopodia and Lamellipodia

Filopodia and lamellipodia are highly dynamic platelet structures whose primary function is cell migration [77]. Due to their nature, they depend on actin activity to form highly branched networks [78]. This remodeling of the actin structure and the consequent change in cell dynamics is one of the most profound consequences of the cellular response to input signals [77,78]. The formation of the actin cytoskeleton is mainly regulated by the Rho family of GTPases, of which RhoA, Rac1, and Cdc42 are responsible for organizing and stimulating the Arp3/2 complex to promote actin nucleation and the growth of branched actin filaments [79,80]. The latter is a process regulated by WASP through the VCA domains [81].

The formation of filopodia and lamellipodia in the platelets is inhibited under conditions of cholesterol depletion [82]. However, the overexpression of phospholipids in the outer layer of the plasma membrane leads to a recovery of actin polymerization under conditions of cholesterol depletion [83].

In cancer, the filopodia and lamellipodia from platelets have a critical role in metastasis. Mammadova-Bach et al. [84] evaluated the effect of Syk and Scr quenching on filipodia formation and platelet spreading. They observed that platelets formed filipodia under normal conditions and adhered rapidly to galectin-3, whereas platelets deficient in Syk or Scr showed decreased adherence to galectin-3 and decreased filopodia formation. They also showed that ATP release and P-selectin exposure in the platelet membrane were decreased in the Syc- and Scr-deficient platelets. They observed a direct relationship between their results and the transendothelial migration of tumor cells and, therefore, with the metastatic capacity [84].

Mammadova-Bach et al. also showed that GPVI-galectin 3 binding regulates the transendothelial migration of tumor cells in vitro and that the release of ATP from dense granules platelet stimulates the process [84].

## 3. Platelet Membrane

### 3.1. Lipid Composition

The platelet membrane viewed under a high-resolution electron microscope has a wrinkled appearance, with many minute folds and randomly distributed openings originating from the open canalicular system [85]. Like any other membrane in the body, it comprises a phospholipid bilayer in which cholesterol, glycolipids, and glycoproteins are embedded [86,87]. The outermost layer of the platelet has a coating of polysaccharides called the glycocalyx; this is the most dynamic site of the platelet, because it is the site of contact with the cellular microenvironment; unlike the rest of the blood cells, it is thicker in platelets [88,89].

Platelet membrane domains are enriched by cholesterol, relatively saturated lipids, and sphingolipids, and are generated according to the rearrangement of lipids in the membrane. Together, these domains act as a functional platform that recruits more lipids and proteins, and can regulate cellular functions [90,91,92].

Lipidomics studies have reported that the lipid composition of the platelet membrane is crucial for the functioning of the cell. The lipid composition of the platelet membrane is responsible for a series of phenomena, such as the shape, curvature, and flexibility of the membrane and the flux of molecules through it [93,94]. It has also been shown that changes in the lipid composition of the platelet membrane induce structural and functional modifications such as activation, degranulation, and exocytosis processes [93,94].

In the following paragraphs, we summarize the most important studies about platelets lipidomic in order of date:

(1) Watanabe et al. carried out a lipidomic study in 1998 [95]. They reported that the primary saturated fatty acids in the platelet plasma membrane are palmitic acid (17%) and stearic acid (21.3%), while the primary unsaturated fatty acids are arachidonic acid (22%), oleic acid (17.1%), linoleic acid (6%), docosahexaenoic acid (2.5%), and eicosapentaenoic acid (2%). The authors concluded that the presence of polyunsaturated fatty acids (PUFAs) in the phospholipids of the plasma membrane reduces the bending stiffness of the membrane and makes it more flexible by reducing the energy required for deformation and fission. PUFAs are related to biological functions in both healthy and diseased organisms, especially in cardiovascular diseases [95].
(2) Skeaff and Holub [96] reported that, during platelet activation, thrombin, a platelet activator, causes a decrease of up to 45% in the content of phosphatidylinositol in the platelet plasma membrane [96].
(3) Lagoutte-Renosi et al. [94] carried out a lipidomics study on resting platelet membranes. Their results showed that the membranes are constituted mainly by phosphatidylcholines (35.2 ± 0.8%), cholesterol (28.35 ± 0.7%), ether-linked phosphatidylethanolamines (10.40 ± 0.2%), sphingomyelins (7.26 ± 0.3%), phosphatidylserine (6.06 ± 0.1%), phosphatidylethanolamines (6.41 ± 0.1%), phosphatidylinositols (2.41 ± 0.03%), and ether-linked phosphatidylcholine (1.84 ± 0.04%) [94].
(4) Cell dynamics studies have shown that the lipid composition in the platelet membrane and, therefore, its access to receptors, is affected by pathologies and certain drugs. In particular, Lagoutte-Renosi et al. [94] reported that ticagrelor is a compound that decreases the fluidity of the platelet membrane by inducing a general stiffness in it. In a lipidomics study, they subjected platelets to previous treatment with ADP and ticagrelor in concentrations of 20 µM. The authors observed an increase in cholesterol from 28.4 ± 0.7% to 30.6 ± 0.5% in the groups that received the treatment in the presence of ticagrelor. When ADP was administered, the reported cholesterol was 27.9 ± 1.2%. In the groups treated with ticagrelor, the phosphatidylcholine content decreased from 35.2 ± 0.8% (the concentration found in the control groups) to 31.7 ± 1.8% [94].

Other studies about drugs’ influence on the composition and conformation of the platelet membrane have been reported, such as the aspirin effect, which modifies membrane proteins’ conformation by reducing membrane lipid fluidity [97]. Lipid-lowering drugs such as Fluvastatin are responsible for the generalized alteration in the lipid composition of platelets, inducing a decrease in the cholesterol/phospholipid ratio of the membrane [98].

Changes in the lipid profile of the platelet plasma membrane have also been described in some pathological states [99]. In patients with alcoholic liver disease, the levels of phosphatidylserine, phosphatidylinositol, palmitic acid, and eicosapentaenoic acid decrease significantly in the platelet plasma [95].

Lorent et al. demonstrated that dietary PUFAs could incrementally affect the stability of lipid raft domains in plasma membranes and create an imbalance between raft domains and coexisting non-raft domains, which in turn can influence signaling events in platelets [100].

Changes in the composition of lipids in the platelet membrane, including phosphatidylcholine and cholesterol depletion, have also been shown in patients with arterial hypertension [101].

In lung cancer patients, Prisco et al. described alterations in the lipid composition of platelet membranes. They found a decrease in phosphatidylcholine and phosphatidylethanolamine, containing linoleic acid, and in esterified n-3 PUFA [102].

In metastasis, the composition of lipids in the platelet membrane plays an important role because (1) the response of platelets in the EMT, (2) the filipodia and lamellipodia formation, and (3) the formation of platelets microparticles (PMPs) are successful, independent of the capacity for fast phospholipid membrane remodeling.

Gasperi et al. confirmed the modulatory influence of ω3 and ω6 (PUFAs) on changes in the fatty acid compositions of cell membrane rafts and how it influences the carcinogenesis process [103].

### 3.2. Lipid Rafts and Signaling

Cholesterol and sphingolipids are crucial elements in the organization and function of the membrane [104]. They are found mainly in specific ordered domains of the membrane and can limit the diffusion rate of proteins within the membrane, depending on the latter’s composition. Both cholesterol and sphingolipids are directly responsible for forming lipid rafts, which are held together by hydrogen bonds; charge pairing; and hydrophobic and van der Waals forces, and are generally surrounded by liquid-phase phospholipids [105,106]. Lipid rafts (LRs) are reversibly formed in dynamic processes, and this dynamic nature allows them to exchange lipid constituents of the membrane or other lipid rafts at any time. They contain proteins that confer signaling properties to cell membranes, as they become functional signal transduction structures that play an essential role in cell activation, endocytosis, cell death regulation, and the development of diseases such as cancer [107,108,109,110].

It has also been reported that the sphingolipids and sterols found in LRs are concentrated in vesicles destined to fuse with the plasma membrane. Specific membrane binding and fusion proteins such as SNARE, SNAP, and SM participate in this process [111,112]. The specialized dynamic membrane microdomain resulting from the fusion of cell membranes is known as a porosome. It has a hole-like appearance with a diameter of 12 to 150 nm, and is responsible for directing exocytosis to specific sites on the cell surface [113,114].

On the other hand, in metastasis, the elevated levels of soluble CD44 in the serum of cancer patients are observed in various human cancers, and can be considered a marker metastasis [115] CD44 is present outside LRs. In human glioblastoma cells, it has been shown to induce MMP-CD44 shedding and tumor cell migration, and has also been reported to colocalize with MMP-9 in LRs, which plays a significant role in tumor invasion [116].

### 3.3. Platelet Membrane Proteins and Receptors

**Figure 2:**
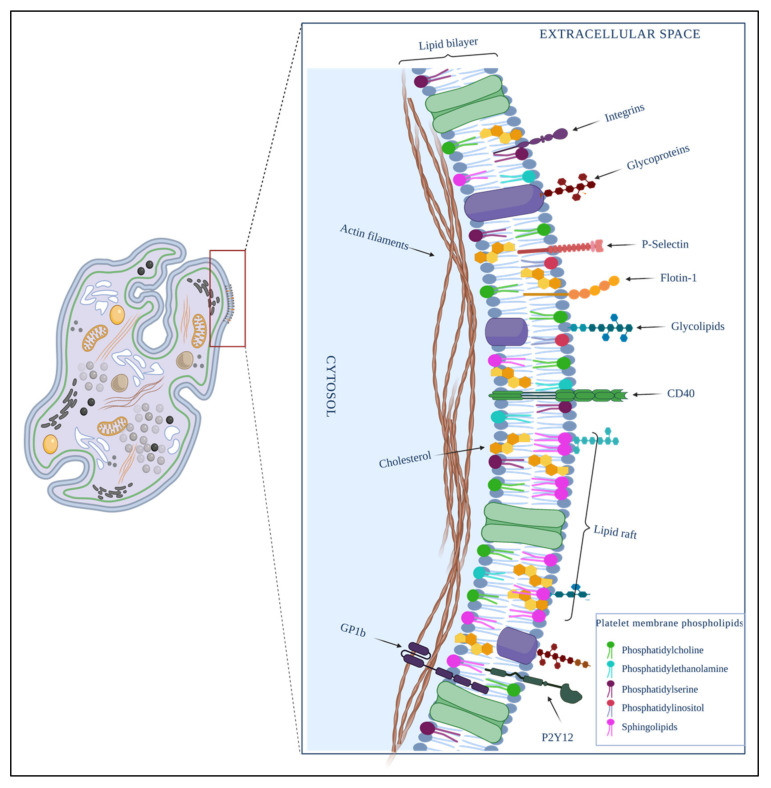
Composition of the platelet plasma membrane. It is constituted by a phospholipid bilayer in which molecular molecules such as cholesterol, glycolipids, and glycoproteins are embedded. The cellular signalization is on a charge of microdomains formed mainly by raft lipids, cholesterol, glycolipids, and proteins such as integrins, immunoglobulins; adhesive glycoproteins; and receptors P-selectin, flotin-1, glycoprotein 1B (GP1B), P2Y12, and CD40, among others. Created by BioRender.

Platelets have several membranes that protect and contain organelles such as mitochondria, granules, OCS, and DTS. There is a different distribution of proteins in each platelet membrane. Many of those proteins act as specific receptors and activate the organelle’s cover functions. For example, proteins such as integrins [45,52], adhesive glycoproteins [52], and receptors of the leucine-rich repeat family, among others [52], are expressed in the outer cytoplasmic membrane of platelets (Figure 2).

Some platelet receptors and transmembrane proteins also play a relevant role in cancer development. It has been proposed that the first interaction between platelets receptors and cancer cells occurs to form a protective barrier around the cancer cell and protect it against anoikis, shear force, and the immune system. This is a spotlight, since several new mechanisms and contributions to metastasis have been attributed to this family of platelet receptors in the last years.

In the following paragraphs, we describe some of them.

(1) Integrins, whose primary function is to maintain platelet adhesion and aggregation during the vascular injury repair response. According to Felding-Habermann et al. [117], integrin αvβ3 supports the breast cancer metastatic phenotype, as this integrin is up-regulated in invasive tumors and distant metastases. They observed that breast cancer cells could exhibit a platelet-interactive and metastatic phenotype that is controlled by the activation of integrin αvβ3.
  Integrin αIIbβIII is mainly expressed in platelets and cancer cells [118,119], although its role is still unknown. Zhang et al. reported that platelets promote the adhesion of melanoma cells to the endothelium in vitro under shear force conditions [120].
(2) P-selectin is an activated platelet receptor that can bind to several human cancer cells such as colon cancer cells, lung cancer cells, breast cancer cells, and gastric cancer cells. P-selectin plays an essential role in metastasis. For example, platelets expressing P-selectin interact with cancer cells in TME and supply various growth factors and mitogens, including platelet growth factor 4 [121].
(3) PAR-1 belongs to protease-activated receptor family-related G protein-coupled receptors activated by the cleavage of part of their extracellular domain. They are highly expressed in platelets, and it has been recently reported that their overexpression is related to invasive and metastatic tumors [122].
  Boire et al. [122] demonstrated that PAR-1 is required and sufficient to promote the growth and invasion of breast carcinoma cells in a xenograft model. Furthermore, they demonstrated that MMP-1 is an agonist of PAR1, cleaving the receptor at the proper site to generate PAR1-dependent Ca2+ signals and migration, so MMP-1 in the stromal-tumor microenvironment can alter the behavior of cancer cells through PAR1 to promote cell migration and invasion [122]
(4) CLEC-2 is another platelet receptor involved in metastasis. Is a transmembrane glycoprotein with the Hemi ITAM (hemITAM) YxxL motif in its cytoplasmatic tail, tyrosine-based activation motifs (ITAM), and hemITAM being very useful in platelet-assisted metastasis [123].

The podoplanin, a transmembrane glycoprotein identified as a surface receptor in cancer cells of various types, is the primary ligand to CLEC-2. The expression of CLEC-2 is mainly restricted to megakaryocytes, platelets, dendritic cells, and Kupffer cells. It has been reported that, by antibodies that block platelet aggregation activity through podoplanin domains, the role of podoplanin domains in platelet aggregation was confirmed in the CLEC-2 binding and tumor emboli formation, concluding that the inhibition of these domains prevents podoplanin-mediated tumor growth and metastasis [123].

Some drugs, such as 5-nitrobenzoate-2CP, inhibit the podoplanin-CLEC-2 union, which causes the inhibition of tumor cell-induced platelet aggregation (TCIPA) and the consequent prevention of tumor metastasis without the risk of hemorrhage [124]. Therefore, the selective blockade of CLEC-2 on the platelet surface and its consequent blockade to bind to podoplanin may provide effective therapy against metastasis and thromboembolic complications.

The platelet membrane proteome has been and will continue to be studied by various research groups due to the relative ease with which it can be studied, but also because of the critical role that platelets have been recently found to play as reporter cells and generators of microenvironments conducive to the development of various diseases, including cancer.

The platelet membrane has been reported to be densely packed with highly specific surface receptors that finely regulate signal-dependent platelet activation [125,126] and can also adaptively regulate the release of granules for coagulation [127], inflammation [128], atherosclerosis [129], antimicrobial host defense [130], angiogenesis [131], wound repair [42], or metastasis [15,132].

Moebius et al. [133] were one of the first research groups to study the platelet membrane proteome. Using SDS-PAGE and nanoLC-MS/MS, they identified almost 300 proteins. Later, Lewandrowski et al. carried out additional studies to identify platelet proteins. Using 1D SDS-PAGE, followed by nanoLC-MS/MS, strong cation exchange (SCX), reverse phase LC-MS, and COFRADIC, they identified 1282 proteins, of which the vast majority belonged to the plasma membrane and platelet organelle membranes [133].

An important topic in the study of platelet membranes is LRs in resting and activated human platelets. It is widely known that lipid rafts are rich in glycosphingolipids, cholesterol, and saturated phospholipids, as well as specific membrane receptors and intracellular signaling proteins [3,91,92,95,96,97,98,99,100,101,102,103,104,105,106,107,108,109,110]. Their signals are involved in cancer development. Due to the way these LRs are formed, their participation is required for platelet activation, mainly by GPVI and signaling processes, the insulin-like growth factor system, and phosphatidylinositol 3-kinase-AKT [109].

In general, the analysis of LRs in cell membranes has benefited from decades of improvement in the techniques used. The best method involves the use of detergents. It has been shown that for both lipidomic studies and membrane proteomics, Triton X-100 (1%) is, in general, the most suitable detergent [134,135].

Among the proteins identified in platelet membrane LRs, stomatin is the main component of the LRs of alpha-granule membranes [136]. The accumulation of stomatin has been reported in released microvesicles after the activation of platelets by thrombin, which means that stomatin had to undergo a translocation [136].

Activated platelets have been reported to express the CD40 ligand (CD40L), also known as CD154 [137], a transmembrane molecule involved in cell signaling in innate and adaptive immunity [138]. An overexpression of CD40L and its receptor CD40, both members of the TNF superfamily, has been reported in the peripheral blood of patients with breast cancer [139].

Sabrkhany et al. [140] conducted the first study to assess the effect of cancer on the platelet proteome of patients with early-stage lung and pancreatic cancer. Using nanoLC-MS/MS analysis, a total of 4384 unique platelet proteins were identified, of which 85 were found to be significantly modified in early-stage cancer compared to the controls. Tumor resection resulted in further proteome changes and the normalization of the expression levels of 81 differently expressed platelet proteins. In addition, the authors reported that the type of tumor depends on the changes undergone by platelets, probably due to differences in the secretome of cancer cells and the tumor location [140].

There are different platelet subproteomes, such as the plasma membrane, microvesicle, and granule; therefore, analyzing them separately would lead us to better understand the role of platelets in healthy and pathological conditions.

## 4. Platelet Activation

Platelet activation involves various reactions and changes in cell dynamics, the cytoskeleton [90], and various groups of proteins associated with the actin filaments strongly expressed in platelets, such as filamin [141], gelsolin [142], cofilin [143], Arp2/3 [144], and capZ [145]. During platelet activation, the structural lipids of the membrane are remodeled, like OCS and DTS, to assume a role in shape changes, propagation and expansion, microvesicle formation, exocytosis, and degranulation [146].

Complex processes take place during platelet activation, including (1) depolymerization of microtubules [147], (2) deformation of the platelet membrane to give rise to lamellipodia, facilitating cell adhesion [22], (3) the massive recruitment of platelets when blood vessels suffer lesions (this recruitment includes a massive activation of platelets, with the corresponding formation of filopodia, that together form a thrombus that allows the lesion to be repaired) [148], and (4) the release of procoagulant molecules and growth factors by platelets to help repair damage to the vasculature [149].

Various stimuli activate platelets in the presence or absence of high calcium concentrations.

One of the activation pathways of platelets in the presence of high calcium concentrations involves the guanine nucleotide exchange factor I, which is regulated by Ca^2+^ and diacylglycerol (CalDAG-GEFI). This pathway activates Rap1b, a GTP-binding protein [150]. Rap1B is a small protein that activates multiple signaling cascades associated with tumor development and progression. It is also involved in cell proliferation, invasion, adhesion, angiogenesis, and metastasis. Its up-regulated expression has been reported in breast cancer cells [151,152]. Rap1B is inhibited at the post-transcriptional level by some tumor suppressor miRNAs, such as miR-518b in squamous cell carcinoma of the esophagus and by miR-139 and miR-100 in colorectal cancer [153]. Wang et al. reported that miR-28-5p acts as a tumor suppressor in renal cell carcinoma by directly inhibiting Rap1B [154].

Rap1b can initiate the formation of an activation complex, where the adapter molecule called RIAM binds to Rap1 and talin [155]. Talin is activated in this complex and binds to the β3 integrin tail through a second interaction site. This binding creates a cleavage between the α and β subunits of integrin αIIbβ3, which causes a change in the conformation of integrin, from folded to extended. In this way, the integrin-binding site on αIIbβ3 is exposed and activated, which allows αIIbβ3 to bind to fibrinogen and vWF [156].

Activated αIIbβ3 can bind to fibrinogen [157], fibrin [158], or vWF [159], and these bonds provide the dominant cohesive force that holds platelet aggregates together. Various proteins, such as β3-endonexin [160], talin [161], kindlin [162], Src [163], Fyn [164], and Syk [165], can bind to the cytoplasmic domains of αIIbβ3.

Integrins are heterodimeric transmembrane receptors that are crucial for transduction signals in the plasma membrane. They participate in many cellular processes that involve a great diversity of proteins on the surface of other cells or in the ECM. They have various functions, such as adhesion and cell migration [166,167]. In general, the functions of integrins are closely related to the progression and development of cancer [168,169]. Integrins are also regulators of endocytosis and exocytosis [170]; they regulate the degranulation of platelet granules, cell−cell communication, autophagy, phagocytosis, and the release and internalization of extracellular vesicles [171]. These processes are described when considering the role played by platelets in the development of cancer and the metastasis process.

vWF is a multimeric glycoprotein present in the alpha granules of platelets, and through its domains A1 and A3, binds to the exposed collagen after a lesion in the wall of a blood vessel. Furthermore, the A1 domain of vWF binds to GPIbα, the receptor of non-activated platelets that forms part of the GPIb/IX/V complex. This binding enhances platelet aggregation at sites of vascular injury [172].

GPVI/FcRγ chain-mediated signaling is crucial for the adhesion of platelets to collagen and platelet aggregation. GPVI is a platelet-specific receptor that belongs to the immunoglobulin family. It consists of two extracellular domains similar to immunoglobulins, a mucin-like stem, a transmembrane region, and a short cytoplasmic tail. Its presence in the alpha granules of platelets has also been reported. GPVI is associated with the FcRγ chain in the platelet membrane, which carries immunoreceptor ITAM for signal transduction [173].

There is also evidence that the ITAM-containing receptor is the link between the Src family kinase and the activation of Syk in human platelets activated by αIIbβ3 [174].

Ephrins EphA4 and EphB1 are platelet surface molecules anchored by a single transmembrane domain. The EphB1 receptor, ephrin B1, clusters with EphA4 and enables changes in the cytoskeleton that support platelet dissemination and increased fibrinogen adhesion, Rap1B activation, and granule secretion [175].

CLEC-2 can mediate platelet adhesion when it is activated by podoplanin. CLEC-2 leads to the phosphorylation of the tyrosine within the Hemi-ITAM motif, a cytoplasmic signaling motif-containing CLEC-2 that requires the complete formation of the phosphorylated Syk-SH2-hemITAM-CLEC-2 complex [176,177].

Let us remember that the first cause of death in cancer patients is metastasis and the second cause is cancer-associated thrombosis. CLEC-2 is an essential molecule in both processes, capable of modulating platelet activation during hemostasis, thrombosis, and tumor metastasis, which is why it has been considered a good candidate for treatments against cancer metastasis and cancer-associated thrombosis. However, podoplanin is also expressed in normal tissues, so it is crucial to analyze its adverse effects [178,179].

PI3K/AKT cannot activate platelets, but can induce the release of platelet granules and amplify signaling to activate platelets via GPIb-IX and ITAM [180].

Platelets self-regulate their activation through negative feedback that counteracts signaling [181]. The immunoreceptor tyrosine-based inhibitory motif (ITIM) and the endothelial cell-selective adhesion molecule (ESAM) are proteins through which platelets negatively regulate the activity of the integrin αIIbβ3 [156].

Molecules such as nitric oxide (NO) [182], prostacyclin (PGI2) [183], and cAMP are capable of inhibiting calcium-dependent platelet activation, while ADP and epinephrine are platelet agonists that induce their activation [184].

## 5. Platelet Extracellular Vesicles

Cells pack and store newly synthesized materials in small vesicles used for their transport to various organelles and the outside of the cells. Synthesized vesicles also allow cell repair damage to any cell membrane by membrane fusion (vesicle−cell membrane) [185] or by normal cellular processes such as fertilization, myoblast formation, and bone homeostasis [186]. Vesicle formation is a mechanism used by virtually any cell as a means of extracellular and intracellular communication and a means of discarding intracellular content [187].

Cancer cells use these exact mechanisms to pack material in vesicles and release regulatory cytokines and biomolecules that aid in developing a TME, metastasis, and angiogenesis [188].

Platelet-derived microvesicles (PMVs), the most abundant in the bloodstream, range in size from 100 to 1000 nm. They account for 70 to 90% of all circulating microparticles and are considered biological platelet activation markers [189]. PMVs contain unique proteins and biomolecules that mediate cellular communication and response, and promote the release of cytokines that participate in inflammatory processes, cancer progression, angiogenesis, metastasis, and tissue regeneration [15].

Microvesicles (MV) and exosomes differ in their origin. While the former comes from budding and direct fission from the plasma membrane, exosomes are generated by the endolysosomal degradation pathway transmitted to the cell surface to fuse with the plasma membrane, and release its contents exosomes to the extracellular environment [190].

Both vesicles and exosomes contain several types of proteins, including cell surface receptors, cytosolic signaling proteins, transcription factors, metabolic enzymes, ECM proteins, RNA-binding proteins, RNA transcripts, microRNAs (miRNA), and genomic DNA fragments [191].

The internalization or fusion of these bodies with target cells is not yet fully understood, but various mechanisms such as endocytosis, phagocytosis, and membrane fusion have been proposed [192,193,194].

PMVs can influence both the microenvironment and the target cell through (1) the activation of receptors found on the cell surface; (2) the transfer of receptors to the cell surface; or (3) direct delivery into the target cell of transcription factors, mRNA, and non-coding RNA, in addition to proteins, cytosines, or growth factors [15,195].

PMVs can deploy receptors as important as CD40 on their surface, which stimulates angiogenic responses in vivo [196]. Platelets can transfer the adhesion molecule CD41 to endothelial cells via PMV, giving them pro-adhesive properties [197]. Through the same pathway, platelets can transfer RANTES molecules to target endothelial cells through mechanisms dependent on GPIIb/IIIa and JAM-A, a process that contributes to the recruitment of monocytes. ICAM-1, the intercellular adhesion molecule 1, can be transferred to endothelial cells by platelets via MV in a PS-dependent process that increases the adhesion and monocyte transmigration [198].

The mechanisms by which PMVs are released involve LIMK, which is phosphorylating cofilin, an enzyme whose function is to cleave filamentous actin molecules, allowing for the assembly and accumulation of actin filaments necessary for the budding of MVs from cell surfaces [199]. ARF6 is another small GTP-binding protein and member of the RAS superfamily whose function is to mediate cytoskeletal remodeling and intracellular vesicle trafficking to the plasma membrane. An increase in ARF6 activity has been observed in breast cancer [200].

Other proteins, such as RAB, function within MVs in intracellular vesicle trafficking processes and exosome release. RAB5 activates PI3K to generate PI3P, which recruits RAB5 to promote endocytic vesicle fusion. RAB7 is also related to the production of exosomes. The absence of RAB7 decreases the ability of the cell MCF7 to release exosomes. While RAB11, RAB27, and RAB35 have been shown to carry MVs to the cell surface, cells begin to accumulate MVs along the inner surface of their plasma membrane in the absence of any of these three proteins, which causes a decrease in their ability to release exosomes [201].

## 6. Cancer Cells

Cancer cells have a cytoskeleton of microtubules, microfilaments, and intermediate filaments made up of tubulin, actin, and numerous proteins, including vimentin and vinculin [202]. The latter has been described as playing a crucial role in the adhesion of tumor cells to substrates. Vinculin is a linker in binding the actin filament (which plays a role in metastasis) to the plasma membrane [203]. The cytoskeleton of cancer cells has several essential functions in developing the disease, namely: (1) allows cancer cells to change shape during TMS, (2) facilitates the adhesion of cells to the substrate, (3) supports chemotaxis processes, and (4) facilitates metastasis [202,203].

Once the tumor has been established and has progressed effectively, the next natural step is to develop metastasis. Metastasis is a process formed by a complex sequence of events involving multiple interactions between cancer cells and other cell types and biomolecules found in the tumor microenvironment, which is delimited by the extracellular matrix. These interactions include, among others, cancer cell−cancer cells, cancer cell−endothelial cell, and cancer cell−platelet interactions [204,205].

Solid tumors have long been associated with thrombus formation, although the pathogenesis of this association has not been fully elucidated and is probably diverse. It is known that tumor cell emboli in the bloodstream are not formed solely by neoplastic cells, but also by platelets and fibrin, with which they form a complex [206]. Rac1 may be responsible for this phenomenon due to the critical role in signal transduction in platelets located in a microenvironment, which allows them to produce a cytoskeletal response and extend their body beyond the geometric limits of extracellular matrices. Rho negatively regulates this process [207].

Cancer cells can also form cell membrane structures in the form of dynamic, actin-rich protuberances, called invadopodia, which give them motility. In addition to the actin core, invadopodia contain other relevant proteins such as contractin, cofilin, N-WASP, Arp2/3, integrins, talins, and vinculins [208]. They also contain various MMP enzymes such as MMP-2 and MMP-9 and proteins of the ADAM family that can degrade the extracellular matrix and penetrate the surrounding ECM stroma and the basement membranes, an essential step in the metastatic process [209]. The activator of these changes in the cell membrane is the initial binding to the ECM [210]. The components of the ECM that trigger the formation of invadopodia include collagen I, collagen IV α1, and collagen XIII α, as well as laminin-111-derived peptides AG73 and C16 [211]. MT1-MMP can initiate invadopodia formation and direct their assembly within the TME [212]. The factors that regulate invadopodia formation within the tumor include growth factors, the EMT, hypoxia, adhesion receptors, chemokines, and degrading enzyme activity [208,209,210,211,212].

The formation of protuberances also takes place on normal cell membranes, called podosomes. These structures have been observed in embryogenesis, wound healing, inflammatory response, and organ regeneration, as in the case of osteoclasts [213].

The main difference between both membrane structures is stability. Invadopodia are more stable as podosomes and for more hours than podosomes [214].

## 7. Contribution of Platelets to Cancer Development

**Figure 3:**
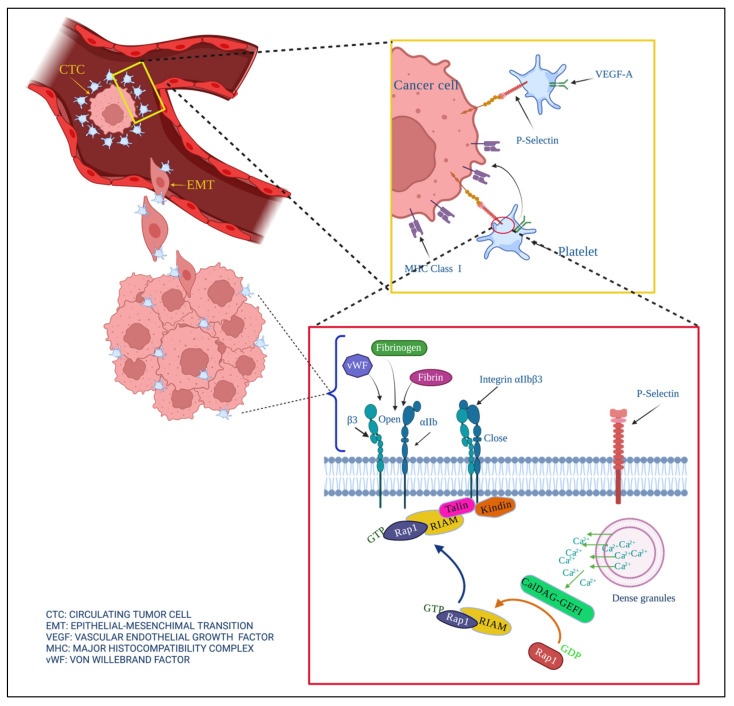
Contribution of platelets in the development of cancer. Cancer cells lose their cell junctions and travel through blood vessels (extravasation), becoming circulating tumor cells (CTC). Platelets help prevent anoikis, help metastatic cells avoid shear force when they extravasate, and confer molecules of the major histocompatibility complex MHC on the tumor surface to prevent their elimination by the immune system.

Interactions between cancer cells and platelets strengthen cancer development, angiogenesis, and metastasis (Figure 3). Their interactions are the reason for hypercoagulation and increased risks of thrombosis in cancer patients.

Platelets found in the tumor microenvironment as a consequence of the creation of new blood vessels are activated and release a series of cytokines, growth factors, and factors, such as VEGF, CCL5, PDGF, TGFβ, PG, TPM3, LPA, PF4, PAF, and HGF, that promote EMT [15].

Metastasis begins when cancer cells invade the tumor’s extracellular matrix and migrate to distant sites through blood or lymphatic vessels after the breakdown of this bordering tissue [15,215]. Migrant cancer cells have previously undergone a process in which they lose their intracellular junctions and apical polarity, changing morphologically towards a mesenchymal type, and acquiring new invasive metabolic and functional capacities. This process is called EMT [216]. Labelle et al. demonstrated that cancer cells have contact with platelets as well as their support in EMT. Another study showed that cancer cells were only generated when platelets were released, and that the gene expression signatures associated with EMT and tumor progression were enriched only in EMT. These same authors demonstrated by electron microscopy that tumor cells that directly contact platelets can engulf parts of them [30].

Another more recent study demonstrated that direct contact between platelets isolated from patients with advanced gastric cancer and gastric cancer tumor cells induced processes of migration, invasion, adhesion, and expression of MMP9 in tumor cells [217]. Extravasated platelet aggregates have been evidenced in invasive parts of clinical samples from human pancreatic cancer biopsies. These aggregates are associated with markers for the first steps of EMT, such as increased expression of Snail1 and reduced E-cadherin. Ishikawa et al. reported that in 60% of the samples from a Japanese cohort of patients with HER2 negative breast cancer (biopsy samples), platelets were found directly surrounding the primary tumor cells, and that these tumor cells showed the expression of EMT markers [217].

Another component that platelets release when interacting with the tumor microenvironment is lysophosphatidic acid (LPA), a lipid with signaling properties similar to those of the growth factor. This acid up-regulates the activity of different matrix metalloproteinases in cancer cells, promoting the detachment of tumor cells from the primary site and their entry into the circulatory system [218].

Thanks to the coating of platelets on the CTCs, the epithelial−mesenchymal transition (EMT) can occur through direct contact between receptors and ligand that are found on the surface of the membrane of both cells, such as P-selectin. Once CTCs have managed to survive in the circulatory system with the help of platelets, they fulfill the objective of creating metastatic foci.

Platelets support the angiogenesis process through factors such as VEGF-A expressed in platelets. CalDAG-GEFI interactions activate Rap1b and bind talin. This binding trigger platelet activation by integrin αIIbβ3, where it can bind fibrinogen, fibrin, or vWF.

For the vasculature to proliferate in the tumor, the proliferation of endothelial cells, which are dependent on VEGF-A, is required. The primary regulator of VEGF-A is platelets. Platelets play a critical role in angiogenesis and the intravasation of cancer cells into the circulatory system [219]. Once cancer cells have reached mesenchymal morphology, their passage between the endothelial layer of the tumor vasculature and the vascular lumen is much easier. This intravasation step is supported by localized and transient TGF-β signaling, and by the expression of EGF by platelets and their receptors in tumor cells [220].

Once cancer cells have managed to enter the bloodstream, platelets are their main defense mechanism against the shear force and the circulating immune system, mainly natural killers [221]. How platelets cover CTC starts with the recognition between receptors and ligands found on the surface of the membrane of both cells [15,218]. P-selectin can recruit platelets to tumors by binding to Talin1, which triggers platelet activation by integrin αIIbβ3 and platelet recruitment. It has been reported that both platelet−endothelial cell adhesion and the formation of metastatic niches depend on integrin αIIbβ3 and P-selectin [121].

Platelets transfer MHC class I molecules to the tumor cell surface via the GARP/TGFß axis to avoid the immune system’s onslaught on the CTC. This coating of CTCs by platelets can form intravascular micro-clots. All the factors conferred by the platelet by CTC provide firmness in cell proliferation and blood vessel formation in the tumor microenvironment [222].

Once CTCs have managed to survive in the circulatory system with the help of platelets, they must fulfill the objective of creating metastatic foci, so they must leave the blood system and seed a second metastatic tumor [217,218,219,220,221,222,223]. Platelets stimulate extravasation by releasing ATP from their dense granules upon activation, which modulates endothelial junctions and the endothelial cytoskeleton to induce a breakdown of the endothelial barrier [223]. Finally, with all the assistance received from the platelets, CTC colonizes the sites of the secondary tumors.

## 8. Concluding Remarks

The role of platelets has remained very far from being only vigilantes of hemostasis, immunity, and repair of tissue damage. Although there are many mechanisms to be known, today, it is known that platelets play a transcendental role in the development of many diseases, including cancer, diabetes, and COVID-19. Despite being very small and enucleated cells, they contain the necessary information in dense alpha granules, lysosomes, and the cytoplasm and the plasma membrane to play critical roles within the organism.

Without a doubt, we believe that platelets still have a lot to tell us and that they will change the paradigms to face the various diseases that afflict humans.

## Figures and Tables

**Table 1:** Proteins and receptors found in platelet membranes and granules membranes [45,46,47,48,49,50,51,52,53,54,55,56,57,58,59,60,61,62].

Platelet Membrane Leaflet		Alpha Granules Membrane Leaflet		Dense Granules Membrane Leaflet	
Inner	Outer	Inner	Outer	Inner	Outer
Cadherins^BC^	GPIIb/IIIa^LC,BC^	APP^CP,PC,BC,LC^	CD62P^CG,BC^	RAB1^LiC,BC^	RAL 20^BlC^
glycoprotein IV^OS^	Integrin^BC,OC^	PDI^BC,OC^	Multimerin^OCC^	RAB 4^PC,LiC,BC^	
CD53^BC,KC,LC^	Fibrinogen^CCR,PC^	PTK^BlC,CCR,BC,LC^	CD3626	RAB 6^BC^	
CD37^LH,AML^	FYN^BC,CCR,OC^	Fibrinogen^PC,CCR,^	GPIb^BC,OC^	RAB 8^BC,OC^	
CDC42^BC,PC,LC^ RANTES^GC,OC^	Fibronectin^BC,PC,LC^ PKC^BC,LC^	PF4^OC,BC^ Serglycin^CE^	GBIIbIIIa factor V^BC^	RAP 1^BC,GC^	
GpIIb/IIIa^LC,BC^	P-selectin^LC,OC,CCR^	PBP^BC,LC,GC,LC^	GMP140^BC,LC,CCR^		
E-selectin^BC,CCR^	Osteonectin^BC,PC,OS,LC^	NAP-2^LC,CCR,OC^	CD63^BC,LC,PC,LiC,CCR^		
Caveolin^BC,CG^	CD36^CC,CCR,BC^	SYK^BC^	VAMP^BC,CCR,LC,PC^		
β-dystroglycan^BC,KC,^	Fibrin^GC,EC,LC,BlC^	Multimerin^CC^	SNAP23^CCR,BC^		
Filamin^OC,CCR^	Myosin^BC,PC,PhC^	Celubrevina^C^	VWF^CCR,BC,LC^		
Gelsolin^BlC,LC,BC^	Cofilin^PC,BC,BlC^	CD72^CCR,BC^	LAMP227^KC^		
Cofilin^LC,P,BC^	RGS^CCR,LIN,LC^				
Sec1^BC,CCR,Ml^	CIB1^OC,KC,EnC^				
NSF^OC,CCR,LiC^	Talina^CCR,PC^				
syntaxins 2/4^CCR^	SHC^BC,GC,CCR^				
Actin^GC,CC,BC^	SRC^OC,OS,PC^				
Calreticulin^GC,BlC,PaC^	PKA^BC,OC,OS^				
Stromatin^BC,LC,CC^	PECAM1^BC,BlC,CP^				
p65^Ml,BC,PC,OC^	CEACAM1^AML,LC,CCR,BC^				
STIM1^BC,CCR^	Calmodulin^PaC,GC,PC^				
TMEM16F^C^	P2Y12^OC,BC,LC,PaC^				
Calmodulin^KC,CCR,PC^	P2Y^CCR,PC,LC^				
LAT^BC,TC,CCR^	P2Y1^OC,PC,GC^				
SLP-76^C^	MAC1^LiC,OC^				
p38^GC,CCR,BlC^	Collagen^CCR,BC,TC^				
DAG^BC,BlCC^	Galectin^CCR,GC,PaC^				
ARF6^LC,OC,TC^	WASP^BC,LC,PC^				
SCAR/WAVE^BC,CCR^	Zyxin^CCR^ GpHb/IIIa^BC^				
	Syntaxin^CC,BC,LiC^				
	SNAP^LiC,CCR^				
	SNARE^CC,BC,GC,CCR^				
	Actomyosin^BC,LC,PC^				
	ARF6^PaC,CCR,KC,LC^				
	SIRT1^OC,PC,BC,LC^				
	SANRE^GC,CCR,PC,CC^				
	GPIb/IX/V^BC,OrC^				
	Vitronectin^AML,CCR,PC^				
	Thromboplastin^LC,LiC,GC^				
	Thrombospondin^OC,CCR,CC^				

Type of cancer abbreviations, where the protein or receptor from platelet has been reported as a key factor: (C) cancer no specified; (BC) breast cancer; (OS) osteosarcoma; (CCR) colorectal cancer; (PC) prostate cancer; (KC) kidney cancer; (LC) lung cancer; (LH) non-Hodgkin’s lymphoma; (AML) acute lymphoblastic leukemia; (GC) gastric cancer; (OC) ovarian cancer; (BlC) bladder cancer; (Ml) melanoma; (LiC) liver cancer; (CC) cervical cancer; (PaC) pancreatic cancer; (LaC) laryngeal cancer; (TC) thyroid cancer; (EC) esophagus cancer; (PhC) pharyngeal cancer; (LIN) lymphoma; (EnC) endometrial cancer; (OrC) oral cancer.

## Data Availability

Not applicable.

## Abbreviations

ADP	adenosine diphosphate
CAF	fibroblasts associated with cancer
CTC	circulating tumor cells
DMS	demarcation membrane system
DTS	dense tubular system
ECM	extracellular matrix
EMT	epithelial mesenchymal transition
ESAM	endothelial cell-selective adhesion molecule
GP1b	Glycoprotein Ib
ITAM	immunoreceptor tyrosine-based activation motifs
ITIM	immunoreceptor tyrosine-based inhibitory motif
LPA	lysophosphatidic acid
LRR	Leucine-rich repeat family
LRs	lipid rafts
miRNA	microRNAs
MMP	metalloproteinases
mRNA	messenger ribonucleic acid
MV	Microvesicles
NK	natural killer cells
NO	nitric oxide
OCS	open canalicular system
PMVs	Platelet-derived microvesicles
PUFAs	polyunsaturated fatty acids
TME	tumor microenvironment
TNF	tumor necrosis factor
vWF	von Willebrand factor
